# Eco-Friendly Greener Synthesis of Nanoparticles

**DOI:** 10.34172/apb.2020.067

**Published:** 2020-08-09

**Authors:** Brahamdutt Bhardwaj, Pritam Singh, Arun Kumar, Sandeep Kumar, Vikas Budhwar

**Affiliations:** Department of Pharmaceutical Sciences, Maharshi Dayanand University, Rohtak-124001, India.

**Keywords:** Nanoparticles, Eco-friendly methods, Polysaccharides, Nano-biotechnology, Antimicrobial activities

## Abstract

The exploitation of naturally obtained resources like biopolymers, plant-based extracts, microorganisms etc., offers numerous advantages of environment-friendliness and biocompatibility for various medicinal and pharmaceutical applications, whereas hazardous chemicals are not utilized for production protocol. Plant extracts based synthetic procedures have drawn consideration over conventional methods like physical and chemical procedures to synthesize nanomaterials. Greener synthesis of nanomaterials has become an area of interest because of numerous advantages such as non-hazardous, economical, and feasible methods with variety of applications in biomedicine, nanotechnology and nano-optoelectronics, etc.

## Introduction

In the current scenario of drug delivery, nanosystems like nanoparticles (NPs), liposomes, dendrimers, solid lipid NPs and others are being employed for a controlled, sustained and targeted delivery of active pharmaceutical entities. All of these nanomaterials have various advantages and patient-friendly because of reduction in dose frequency and much better retention time of drugs within the targeted site compared to conventional dosage forms. The primary aim of these nanosystems is to sustain the therapeutic amount of drug within the bloodstream for a longer time period. But still, there are some important factors that affect the delivery of drugs as the drug carrier, targeted site for delivery of drugs, drug administration route and the tactic considered to boost therapeutic efficiency of medication. These factors reduce the undesirable effects of the active pharmaceutical entity and improved the therapeutic performance of drugs.^1^ Although UV irradiation, aerosol technologies, lithography, laser ablation, ultrasonic fields, and photochemical reduction techniques have been used successfully to produce NPs, they remain expensive and involve the use of hazardous chemicals, which leads to major attention toward the expansion of eco-friendly and sustainable greener synthesis of NPs.^2^ Nano-biotechnology is a newer term formed through merging of three different fields i.e. nanotechnology, microbiology and biotechnology as microbes are being used for synthesis of nanomaterials through biotechnological methods. Bioremediation and bioleaching bio-mineralization have been performed through metal–microbe interactions, but nano-biotechnology is at its early stage period. In spite of their potent outcomes, it carries an encouraging application in drug delivery through nano-methods. This review article highlights the green synthesis of NPs from various sources such as plants, polysaccharides and microbes with their applications in different areas.^3^

## Why green methods for synthesis of nanoparticles?

**Figure 1:**
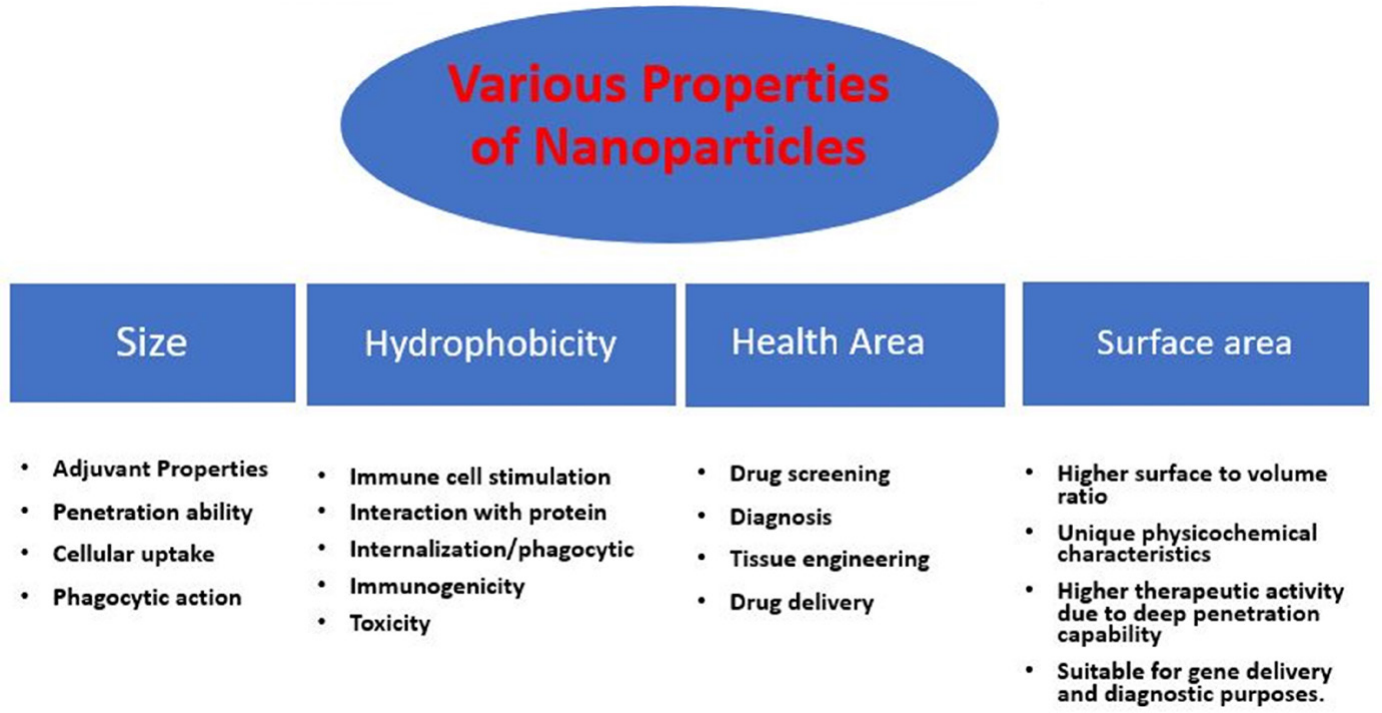
Different properties of nanoparticles.

Currently, there are numerous chemical and physical methods available in the literature for production of nanomaterials, which deliver a higher rate of production and well-controlled size and shape of nanomaterials but these approaches are discouraging due to higher loss of energy and capital, use of noxious chemicals, and production of large amount of bio-waste. These key factors influence the commercial level scale-up process of nanomaterials economically as well as environmentally. Additionally, the clinical use of nanomaterials prepared through chemical methods has been limited due to issues of biocompatibility, toxicity and stability. These components elevates requirement of eco-friendly, cheaper and biocompatible methods for production of nanomaterials. In comparison to conventional physical and chemical methods, greener route for NPs synthesis offers economical, environment-friendly and nontoxic approaches (Figure 1).^3^

## Nanoparticles synthesis from plants and their extracts

Due to environment friendly behavior, lower toxicity, cheap, more biocompatibility and better size controlling aspects offered a higher prominence for the production of nanomaterials through greener ways over physical and chemical methods. The primary goal of nanotechnology is to develop a reliable and better production method, which regulates the chemical composition, morphology and better monodispersing systems in large scale production of nanomaterials. Numerous eco-friendly methods for synthesis of NPs systems from plants, bacteria, and fungi have been recommended in literature because of their economical, low toxicity profile and biocompatible in nature. Jayprakash et al, prepared silver NPs (AgNPs) with *Tamarindus indica* natural fruit extract through microwave-assisted greener synthesis. The plant-based extract was acted as a reducing as well as capping mediator for AgNPs synthesis. Morphological characterization of NPs was performed using different techniques such as X-ray diffraction (XRD), high-resolution scanning electron microscopy and transmission electron microscopy. The average particle size of prepared NPs was found to be 6-8 nm and XRD studies revealed the face-centered cubic silver presence. Good antibacterial action was exhibited by the prepared silver NPs through a simplistic, economical and greener method. AgNPs production methods using plant extracts are reported in literature like *Mangifera indica* leaf, *Murraya koenigii* leaf, *Jatropha curcas* , *Mangosteen* leaf, *Cinnamomum zeylanicum* leaf, *Aloe vera* , *Camellia sinensis* , honey and mushroom. Fruit extracts were also being utilized for NPs preparation such as lemon, pear, papaya, goose berry and tansy etc. NPs prepared through consuming fruit or plant extracts offer an advantage of non-aggregation of NPs over long term storage conditions.^4^

*Aloe vera* plant extract was used for the synthesis of spinal shaped polycrystalline nanopowders of Ni_x_Cu_0.25_Zn_0.75–x_Fe_2_O_4_ (where x = 0.25, 0.35, 0.5) having an average particle size of 15-40 nm via simple solution method consuming metallic nitrates and *Aloe vera* plant extract mixture. Ferromagnetic activities were exhibited from obtained nanomaterials.^5^ Coffee and tea extracts had been exploited for synthesis of stable NPs of noble metals (i.e. Pd and Ag) in the size range of 20-60 nm. These stated approaches might be employed for NPs production of other noble metals like Pt and Au.^6^ Oxides of various metals had been utilized for nanoparticle production like titanium oxide. *Nyctanthes* leaf extract and titanium isopropoxide solution were used for obtaining titanium (IV) oxide nanoparticle having average size (100–150 nm).^7^ Aqueous extracts of the manna of *Hedysarum* plant and the soap-root (*Acanthophyllum bracteatum* ) plant were exploited to prepare the NPs and an average diameter of the prepared NPs in solution was about 29–68 nm (Table 1).^8^

**Table 1:** Nanoparticles prepared from plants and their extracts^8-25^

**Source**	**Plant part used**	**Nanomaterial**	**Application**
*Acanthophyllum bracteatum* plant	Aqueous extracts of the manna of *Hedysarum* plant and the soap-root	Silver Nanoparticles	Antibacterial activity
*Aloe barbadensis* Miller	Leaf extract	Zinc oxide NPs	Antimicrobial, and dermatologic application
*Plectranthus amboinicus*	Leaf extract	Zinc oxide NPs	Photocatalytic activity
*Garcinia xanthochymus*		Zinc oxide nanoparticle	Antioxidant activity, photocatalytic activity
Honey		Zinc oxide nanopowder	Cytotoxicity effects
*Lycopersicon esculentum* Mill.	Leaf extract	Silver and gold NPs	Antimicrobial activity
*Artocarpus heterophyllus* Lam.	Fruit extract	Gold NPs	Antimicrobial activity
*Trianthema decandra*		Silver and gold NPs	Antimicrobial activity
*Helianthus annuus*	Flower	Gold NPs	Antimicrobial activity
*Saraca indica*	Bark extract	Gold NPs	Catalytic reducing agent
*Phyllanthus amarus Schum and Thon*	Aqueous leaf extract	Silver and gold NPs	
*Azadirachta indica*	Leaves	Silver NPs	Biolarvicidal
*Catharanthus roseus*	Leaves	Palladium NPs	Catalytic activity in dye degradation
Banana	Peel	Cadmium sulphide	
Red ginseng	Root	Silver NPs	Antibacterial
*Cocos nucifera*	Leaves	Lead NPs	Antibacterial and photo- catalytic activity
*Citrus medica*	Fruits	Copper NPs	Antimicrobial
*Abutilon indicum*	Leaves	Silver NPs	Antibacterial

## Nanoparticles synthesis from natural polysaccharides

In spite of plant parts extracts polysaccharides are also being employed for nanomaterials preparation as an eco-friendly approach. Sulfated polysaccharides obtained from marine red algae (*Porphyra vietnamensis* ) were utilized for silver NPs synthesis. The particle size of prepared nanoparticle was found to be about 13 ± 3 nm and surface plasmon resonance centred at 404 nm. The spectroscopic study revealed the connection of reduction of silver nitrate by sulfate moiety of obtained polysaccharide.^26^ The Greener method for preparation of silver NPs was employed by dissolving silver (III) ion-containing rice wine and soda over-temperature raging (25-55°C) at pH 6.5 without using extra protective material. In this technique, rice wine played dual role as solvent and reducing agent while soda was utilized as base catalyst and protective agent. The obtained mixture exhibited higher stability and negligible precipitation even after long term storage for months.^27^

In another study, Chen et al proposed deformable liposome of flurbiprofen coated with chitosan for ocular drug delivery to improve the transcorneal absorption and enhanced the pre-corneal drug residence time. These liposomes were formulated through the modified ethanol injection technique and then chitosan was coated over them. Gamma scintigraphy technique was employed to check the pre-corneal retention period and draining out dynamics of drug *in-vivo* . The deformable liposome of flurbiprofen coated with chitosan prolonged the area under the remaining activity-time up to 2.84 and 1.53-fold compare to flurbiprofen solution and deformable liposome of flurbiprofen respectively. No ocular injury or irritation was reported with use of deformable liposome of flurbiprofen coated with chitosan *in-vivo* .^28^ Curcumin, N,O-carboxymethyl chitosan and oxidized alginate-based *in situ* injectable nanocomposite hydrogel formulation showed a novel dermal wound dressing application. The development of nanocomposite of curcumin involved incorporation of methoxy poly(ethylene glycol)-β-poly(-caprolactone) copolymer into N,O-carboxymethyl chitosan and oxidized alginate hydrogels system. Prepared hydrogels were injected on rat dorsal injuries to study the healing process. The study revealed the considerable improvement in epidermal re-epithelialization and deposition of collagen within the tissue of wound.^29^ In 2012, Tian et al, prepared glycyrrhetinic acid and modified sulfated chitosan-based drug carrier system for anticancer activity. The prepared drug-carrying nanosystem was found to be spherical in shape and around 200 nm in size, showing a significant anticancer activity.^30^ Among the various biological NPs, those produced by medicinal plants have been found to be the most pharmacologically active, possibly due to the attachment of several pharmacologically active residues (Table 2).

**Table 2:** Nanoparticles prepared from natural polysaccharides^31-44^

**Material used**	**Drug**	**Nanomaterial**	**Uses**
Alginate	Isoniazid and pyrazinamide	NPs	Anti-tubercular activity
Alginate–oligochitosan–Eudragit L100-55	Naproxen	Microparticles	Non-steroidal anti-inflammatory activity
Sodium Alginate	Isoniazid	Microspheres	Anti-tubercular activity
Chitosan	Zinc sulphide and mannose	Nanoprobes	Targeted cancer imaging
Galactosylated chitosan	Doxorubicin	Microbubbles	Anticancer activity
Chitosan	Prednisolone	NPs	Renal targeting drug delivery
Lauryl succinyl chitosan	Human insulin	Micro/nano-particles	Oral peptide delivery system
Hyaluronic acid	Tacrolimus	Niosomes	Ocular drug delivery
Alginate	Cisplatin and doxorubicin	Liposome	Anticancer drug delivery
Chitosan	Artemisinin	Magnetic NPs	Drug delivery in breast cancer cell
Chitosan	IR820- iron oxide	Magnetic nanosystem	Imaging agent against melanoma
Gelatine	Dextran sulphate	NPs	Expression of MUC5AC in ocular surface epithelial cells
Gum cordia	Fluconazole	NPs	-
Cationized gelatine	Dextran sulphate and chondroitin sulphate	NPs	Ophthalmic drug delivery

## Nanoparticles synthesis from microbial origin

Plant-based extracts and microbial cultures have been used for the greener or eco-friendly synthesis of NPs all over the world. Due to quick growth rate, low-cost cultivation and capability of survival in ambient environmental conditions like temperature, pressure and pH make microbes a favorable candidate for NPs synthesis. These have inherent potential to prepare NPs of inorganic materials via reduction mechanism through intracellular and extracellular routes because of their survival capability in the metallic noxious surroundings. Metallic ions present in the environment are trapped by microbes and with the help of enzymatic activity and microbes convert these ions into their elemental forms.^3^

Fungi based greener synthesis of nanomaterials is attaining much popularity worldwide.^45^ In comparison to bacteria, higher yield of NPs is obtained using fungal strains, because of larger biomass. NPs with different shapes and sizes were prepared by using numerous fungal species such as *Fusarium oxysporum* , *Verticillium luteoalbum* , *Trichothecium* sp., *Colletotrichum* sp., *Alternata alternate, Aspergillus oryzae* , *Trichoderma viride,* etc.^46^ Largely, the use of toxic or hazardous chemicals can be eliminated for production of biologically and pharmaceutically important materials by the use of eco-friendly greener chemicals and microorganisms. Numerous reports have been published for greener synthesis of metal oxide NPs (like manganese oxide, copper oxide, iron oxide, titania) with the use of microorganism’s cultures like *Lactobacillus* sp., *Yeast cells, Fusarium oxysporum, Shewanella oneidensis, Saccharomyces cerevisiae* and *Bacillus* sp. cells etc.^45^ Metallic ions felt great reduction effect over them due to bacteria leads to synthesize NPs. Research studies revealed the bacterial based reduction mechanism over metallic ions leads to precipitation of metals to nanometres scale. Fungal species had different enzymes (intracellular and extracellular) which could produce a well-defined size and shaped mono-dispersed NPs.^46^

In a study, Malarkodi et al, biosynthesized NPs of titanium dioxide using *Planomicrobium* sp. and their anti-microbial activities were estimated against *K. planticola* , *Bacillus Subtilis* and *Asper niger* .^47^ NPs of iron were prepared using *Fusarium oxysporum* presenting antimicrobial activity against *Escherichia coli, Staphylococcus* sp. and *Bacillus* . The respiration mechanism of microbes depends on concentration of substrates, was restricted by these iron NPs via limiting the oxygen supply.^48^ The concentration of substrates, pH and temperature of the incubated medium influenced the growth, mono-dispersion and dimensions of the formulated NPs.^49^ In a similar study, Sharma et al revealed that the capping agent and incubation time period directly influenced the stability and size of formulated NPs, respectively.^50^ Synergistic action of different antibiotics viz. nitrofurantoin, ciprofloxacin and carbenicillin with silver NPs prepared via eco-friendly method from *R. stolonifer* were exhibited against ESBL-strains of *Enterobacteriaceae* . Both ciprofloxacin and Carbenicillin exhibited increment of 30.53% and 33.56% respectively, while around 50% of increment was reported with nitrofurantoin.^51^ In the similar fashion, combination of silver NPs prepared from *Brevibacterium frigoritolerans* with various antibiotics (like penicillin G, novobiocin, oleandomycin, vancomycin, rifampicin) improved the antimicrobial effect of these antibiotics especially against pathogenic strains of *Bacillus cereus, Escherichia coli, Salmonella enterica, Vibrio parahaemolyticus, Candida albicans* and *Bacillus anthracis* .^52^

Breast cancer malevolence is one of the major causes of death among women. According to the reports described in literature, these microbes based metallic NPs are offering significant anticancer activity. Platinum NPs biosynthesized from *Saccharomyces boulardii* tested against A-431 and MCF-7 cell lines exhibiting anticancer activity.^53^ Silver NPs prepared using *Cryptococcus laurentii* present a better anticancer effect against cancerous cell line especially breast cancer cell lines. The stimulation of apoptosis, sustainability and endocytic action of tumor cell lines were affected by greener synthesized silver NPs. The endocytic activity of tumour cell was found to be equivalent to efficiency of silver NPs.^54^ Selenium is trace element with anticancer activities and *Streptomyces bikiniensis* was utilized to biological preparation of selenium nanorods exhibiting antitumor activity against MCF-7 and Hep-G2 cancer cells. Deployment of copper bound to chromatin trailed by pro-oxidant effect leads to decrease Hep-G2 and MCF-7 cells and this was the mechanism of action that followed by these nanorods.^55^ *In vitro* anticancer activity against breast cancer and human liver cells viz. MCF-7 and HEPG-2, respectively, were conducted with gold NPs synthesized from *Streptomyces cyaneus* revealing stimulation of mitochondrial apoptosis and cytokinesis detention lead to DNA impairment.^56^ Gold NPs synthesized with *Candida albicans* were estimated to analyze the cancer cells of liver through attachment of NPs with surface-specific antibodies of liver cancer cell. These NPs bounded antibody attached clearly with superficial antigen of affected cell and could recognizably differentiate cancer cell from normal cells.^57^ The use of microbially synthesized nanomaterial in diagnostics is at its initial stages and further research in this area would provide more feasible perspective for future.

Fungal species *Fusarium oxysporum* released a bioactive material via silver nitrate reduction extracellularly. An admirable anti-inflammatory and antibacterial activity are unveiled by silver NPs helps in improvement of wounds healing process. The fungal culture released protein which help in stabilization of silver NPs and nitrate dependent reductase enzyme and quinine shuttle reduce the metallic ions. The antibacterial action of silver NPs prepared by the above-discussed method was evaluated on silk and cotton cloths against *S. aureus* .^58^ Similarly, algae released their protein which not only reduce the silver ions, but also the NPs and thus stabilized the silver NPs. The protein released by *Chlorella vulgaris* played a double role through reduction of silver ions as well as controlling the synthesis and morphology of NPs. The -OH and -COOH groups present in tyrosine and Asp/Glu residues helped in reduction process of silver ions. The metabolites of marine algae-like *Chaetoceros calcitrans, Chlorella salina, Isochrysis galbana* and *Tetraselmis gracilis* reduced the silver ions and thereby synthesized the Silver NPs (Table 3).^59^

**Table 3:** Nanoparticles Prepared from Microbial Sources^60-92^

**Microbial culture used**	**Type of nanoparticles**	**Size of nanoparticles**	**Morphology**
*Aspergillus flavus*	Silver	100 nm	Spherical
*Aspergillus fumigatus*	Silver	10-25 nm	Spherical
*Brevibacterium casei*	Silver	10-50 nm	Spherical
*Fusarium oxysporum*	Silver	15-50 nm	Spherical
*Cladosporium cladosporioides*	Silver	<100 nm	Spherical
*Brevibacterium casei*	Gold	<50 nm	Spherical
*Trichoderma viride*	Silver	3-5 nm	Irregular
*Verticillium sp.*	Silver	<50 nm	Spherical
*Plectonema boryanum*	Gold	<25 nm	Cubic
*Plectonema boryanum*	Gold	<6 µm	Octahedral
*Pseudomonas aeruginosa*	Gold	30 nm	Irregular
*Rhodococcus sp.*	Gold	<15 nm	Spherical
*Shewanella Algae*	Platinum	5 nm	Irregular
*Enterobacter sp.*	Hg	<5 nm	Spherical
*Fusarium oxysporum*	Alloy of silver and gold	<15 nm	Spherical
*Desulfovibrio desulfuricans*	Palladium	<50 nm	Spherical
*Yarrowia lipolytica*	Gold	<15 nm	Triangle
*V. luteoalbum*	Gold	<100 nm	Irregular
*Ureibacillus thermosphaericus*	Gold	<100 nm	Irregular
*Escherichia coli*	CdTe	2-4 nm	Spherical
*Lactobacillus sp.*	BaTiO_3_	<100 nm	Tetragonal
HSMV-1	Fe_3_O_4_	<100 nm	Bullet shaped
*Shewanella oneidensis*	Fe_3_O_4_	<50 nm	Rectangular
*Fusarium oxysporum*	BaTiO_3_	<5 nm	Spherical
Yeast	FePO_4_	<100 nm	Nanopowder
*Fusarium oxysporum*	TiO_2_	<15 nm	Spherical
*Lactobacillus* sp.	TiO_2_	<35 nm	Spherical
*Aeromonas hydrophila*	ZnO	55-75 nm	Spherical
*Fusarium*	ZrO_2_	3-11 nm	Spherical
*Fusarium oxysporum*	CdS	<20 nm	Spherical
*Rhodobacter sphaeroides*	CdS	<10 nm	Hexagonal
*Rhodopseudomonas palustris*	CdS	<10 nm	Cubic
*Rhodobacter sphaeroides*	PbS	<10 nm	Spherical
*Desulfobacteraceae*	ZnS	<5 nm	Bio-Film
Prokaryotes	Fe_3_S_4_	<100 nm	Irregular

## Enzyme-mediated and protein-mediated synthesis of nanoparticles

Biological systems could be used for greener synthesis of NPs in terms of their unique shapes and sizes in a controlled manner. Rangnekar et al prepared gold NPs by using pure α-amylase. In the similar fashion, EcoRI, an endonuclease having free cysteine, reduces the gold ions, while other enzymes were unable to reduce the chloroauric acid to gold NPs without free cysteine exposure.^93^ In another study, Roy et al investigated the capacity of cysteine as a reducing agent in spite of the role of cysteine as a capping material on gold NPs. Various analytical techniques were utilized to investigate the linkage of cysteine with gold NPs like ultra-violet visible spectrophotometry, Fourier transform infrared spectroscopy, XRD and Raman spectroscopy.^94^ Sharma et al carried out a study of gold and gold: platinum NPs synthesis by using urease enzyme as reducing agent. They investigated the role of cysteine in NPs formation. They modified the cysteine in urease by its reaction with 5,5′–dithiobis in non-denaturation conditions. Due to this modification, there was no NPs formation occurred. Patela et al prepared Glycine max’ (soybean) leaf extract mediated palladium NPs. In this study, the protein present in leaf extract acts as reducing agent for formation of palladium NPs. The possible reaction of tyrosine with palladium ions leads to the donation of electron and conversion of palladium to palladium NPs.^95^ Similarly, the glucose oxidase interaction with palladium leads to the formation of palladium NPs. Selenium NPs were produced by using α-amylase from *Bacillus methylotrophicus* but unfortunately, no mechanism behind the study was discussed (Table 4).^96^

**Table 4:** Nanoparticles prepared from various natural sources having antioxidant properties^97-108^

**Species Name**	**Part of species used for extraction**	**Medium used for extraction**	**Antioxidant Properties**
*Cassia occidentalis*	Seeds and Leaves	Methanol	Ferric reducing antioxidant activity
*Terminalia chebula*	Leaves	Ethanol	Increased free radical scavenging potential
*Schotia latifolia*	Stem bark	Aqueous	Free radical scavenging activities
*Pistacia integerrima*	Leaf gall extracts	Ethanol	Higher content of total phenolics and flavonoids found in the ethanolic extract was directly associated with higher antioxidant activity
Poly(acrylonitrile-butadiene-styrene)		Chloroform	Scavenge free radical
*Xanthomonas campestris* produce Xanthan polymer	Bacteria		Antioxidant properties
*Acetobacter xylinum* produce Cellulose polymer	Bacteria		Reducing power
*Sinorhizobium meliloti* produce Curdlan polymer	Bacteria		Antioxidant properties
*Leuconostoc mesenteroides* produce Dextran polymer	Bacteria		Antioxidant properties
*Cystoseira barbata*	Seaweed	Aqueous	*Cystoseira barbata* based alginate polymer exerted moderate antioxidant activity
Bacterial nanocellulose	Cellulose based membrane loaded with caffeic, ellagic and gallic acids	Aqueous	Higher antioxidant properties
*Ficus glomerata*	Leaf gall extracts	Aqueous and methanol	Enhanced antioxidant properties of methanolic extract comparative to Aqueous extraction.

## Shortcomings in green synthesis of nanoparticles

Though microbes offer a safe, eco-friendly and economically viable approach for synthesis of NPs as compared to their chemical alternates, lack of monodispersing system, uncontrolled size, and time-consuming production process and these disadvantages have limited their use on commercial scale. Owing to nontoxicity of biosynthesized NPs, they showed propitious potential in nanomedicine yet their use in drug delivery and diagnostics is at its infancy.^3^ The toxicity of natural polysaccharides could be assessed by 3-(4,5-dimethylthiazol-2-yl)-5-(3-carboxymethoxyphenyl)-2-(4-sulfophenyl)-2Htetrazolium (MTS) and 3-(4,5-dimethylthiazol-2-yl)-2,5-diphenyltetrazolium bromide (MTT) assays, which are currently widely applied since they allow to assess the effect of chitosan NPs onto the cell metabolic activity.^109^

De Campos et al assessed the toxicity profile for chitosan via simple colorimetric assay with tryptan blue dye. The study revealed that up to 2 mg/mL of chitosan concentration exhibited no toxicity. The higher concentration of chitosan may be hazardous for survival of cells but, some scientists claimed that acetate buffer solution (pH 6.0) might contributes to the toxicity for cell survival.^110^ On the basis of MTT assays, some of the reports considered cytotoxic behavior of chitosan NPs were greater in macrophages than in fibroblasts. The higher concentration of particles caused cells death by modifying the metabolism process of cell via nanoparticle internalization, but not due to membrane degradation.^111^

In the case of plants, the charge present over various phytochemicals got changed due to change in pH, which caused the changes in their capability of binding and metallic ions reduction mechanism during synthesis of NPs, affecting the production and morphological characters of NPs. The gold NPs of *Avena sativa* were prepared in large quantity at pH 3.0-4.0 while a bunch of NPs was observed on pH 2.0. The process of aggregation dominated the reduction mechanism of metallic ions, in case of low pH range.^112^

Fungal cultures are extensively being used for the eco-friendly production of nanomaterials. Due to greater quantity of bioactive material secreted by fungi, these were much preferred for large scale production of NPs.^58^ But there are some drawbacks regarding fungi-based NPs production as laborious, time-consuming and costly intensive down flowing process, so for commercial-scale production, cheaper and economical method will be needed. While in the case of bacterial based synthesis methods on large scale, the requirements of hazardous chemicals are low but process of bacterial culturing is laborious and control on the nanoparticle’s morphological parameters is less.^46^ Organized and meaningful studies are required for understanding some of the mechanisms involving in various reactions to find a more well-defined outcome. There were numerous concepts regarding reduction of Ag^+^ to Ag^0^ and the bacteriostatic activity of silver NPs.^2^

## Finding of study

The study reports found green NPs synthesis as far as physical and chemical methods are concerned to be considered much more effective and environmentally friendly. Due to its diverse characteristics, flexibility, various benefits and applications for humans, NPs are one of the most essential and versatile materials. Green sources are a stabilizing and reducing agent for the synthesis of controlled-size and shape NPs. The application of NPs to crops in general increases agricultural growth and yield. As a constant increase in demand for food, there is a low yield for a staple crop. It is therefore important for sustainable agriculture to market metal oxide NPs. During various processes, such as bioimaging, drug delivery, biosensors and gene delivery, the biomedical applications in this field are being stepped up daily. NPs can serve as intelligent weapons against multiple drug-resistant microorganisms and can replace antibiotics in terms of their toxicity properties. This study is intended to further streamline research in this area on novel analytical and clinical associations.

## Conclusion

In summary, here we have discussed various biological or eco-friendly green synthesis of nanomaterials and their biomedical applications. Though, the physical and chemical methods for production of nanomaterials are available currently biological methods are preferred because of their non-hazardous nature as compared to chemical methods. Some of the key factors (like expensive chemicals, higher energy consumption and toxicity) cause the chemically produced nanomaterials unfavorable for use. Thus, a need for biocompatible, greener and economical approaches arises for production of NPs. Plants based extracts, naturally obtained polysaccharides and microbes are the targeted materials for fulfilling the desire of suitable methods for biological production of NPs. But still some numerous concepts are required to be probed in more details like methods for large scale production with cheaper cost and controlled behavior. Detailed investigations regarding controlled morphology, biocompatibility and pharmacokinetic studies are also desirable. So, more research work should be focussed on understanding the concepts and mechanisms involved in biological and economical production of nanosystems using plant sources and microorganisms.

## Ethical Issues

Not applicable.

## Conflict of Interest

The authors have no conflict of interest.

## Acknowledgments

The authors thank the Department of Pharmaceutical Sciences, M.D. University for providing the necessary facilities.
